# Additional dexamethasone in chemotherapies with carboplatin and paclitaxel could reduce the impaired glycometabolism in rat models

**DOI:** 10.1186/s12885-017-3917-x

**Published:** 2018-01-16

**Authors:** Yanxiu Guo, Haoxia Zeng, Xiaohong Chang, Chaohua Wang, Heng Cui

**Affiliations:** 1 Center of Gynecologic Oncology, Peking University People’s Hospital, Beijing, 100044 China; 2 Obstetrics and Gynecology Department, Beijing Chaoyang Hospital, Capital Medical University, Beijing, 100020 China; 3 Department of Obstetrics and Gynecology, Peking University People’s Hospital, Beijing, 100044 China

**Keywords:** Glycometabolism, Chemotherapy, Dexamethasone, Carboplatin, Paclitaxel

## Abstract

**Background:**

Side-effects have been considered as the limitation of the chemotherapy agents’ administration and life quality in patients with ovarian cancers. In order to explore the influence of the chemotherapy agents commonly used in ovarian cancer patients on the blood glucose metabolism in rat models, we conducted this study which simulated the conditions of clinical protocols.

**Methods:**

Eighty clean-grade female Wistar rats were randomized into 8 groups: Group 1 (Negative control), Group 1′ (Dexamethasone), Group 2 (Carboplatin), Group 2′ (Carboplatin-plus-dexamethasone), Group 3 (Paclitaxel), Group 3′ (Paclitaxel-plus-dexamethasone), Group 4 (Combined therapy), Group 4′ (Combined-therapy-plus-dexamethasone). On day 0, 4, 7 and 14, after fasted for 12 h, the rats in all groups underwent a glucose load and their blood glucose, glucagon and insulin levels were measured.

**Results:**

The glucose levels in group 2, 3 and 4 at 1 h after the loading on day 4 significantly increased (*P* = 0.190, 0.008 and 0.025, respectively). The glucagon levels in group 3 and 4 showed a similar trend and the increase was not suppressed by the glucose loading (*P* < 0.001). A significant decrease of insulin levels in group 2, 3 and 4 were observed on day 14 after treatment (*P* = 0.043, 0.019 and 0.019, respectively). The change of HOMA2 %B, an index reflects the ability of insulin secretion was negatively corresponded to the glucose levels, and the trends of HOMA2 IR, an index shows insulin resistance, were positively correlated to the glucose levels. The application of dexamethasone could reduce the degree of increased glucose levels significantly in group 2, 3 and 4. There were no differences in overall survival between the 8 groups. Edema in the stroma of pancreases was observed in group 3, 3′, 4 and 4′ on day 4 after treatment (*P* = 0.002, 0.002, 0.000 and 0.000 respectively) and lasted until day 14.

**Conclusions:**

Carboplatin and paclitaxel administration could cause a transient hyperglycemia in rats. This effect might occur by the combination of glucagon accumulation due to the decrease in islet cell secretion. The additional dexamethasone in the combination protocol of carboplatin and paclitaxel seemed to reduce the impaired blood glucose metabolism.

## Background

Hyperglycemia occurs in 7.9–37% of patients after combined chemotherapy [1–5]. Recently, the combination of carboplatin plus paclitaxel for the treatment of ovarian cancer has received considerable attention [1]. Typically, 6.4–27% of patients on a paclitaxel-containing regimen develop grade 3–4 hyperglycemia (sugar levels greater than 250 mg/dl) [1, 2]. We have discovered that 12.4% of patients with hyperglycemia and 14.9% of patients who first diagnosed diabetes mellitus after 3–9 cycles of chemotherapy among ovarian cancer patients [6]. We compared the different chemotherapy regimens our patients received. Among patients receiving coordinated chemotherapy with paclitaxel and cisplatin or carboplatin, the occurrence of DM was higher. In that study, we hypothesized that chemotherapy may induce diabetes mellitus among patients with malignant gynecological tumors and be one mechanism that interferes with insulin function [6].

Some of the side-effects of chemotherapy have been studied and understood. Hyperglycemia, as one of side-effects of many chemotherapy treatment, becomes more common these days. Little is known about the mechanisms of it. Most reports did not discuss with the mechanism of hyperglycemia after chemotherapy. Some authors concluded part of adjuvant chemotherapy regimens produced an appreciable incidence of hyperglycemia [7].

In 1982, Goldstein et al. elucidated the effects of cisplatin on carbohydrate tolerance and insulin and glucagon secretion in rats [8]. They found the appropriate immunoreactive insulin response to a glucose stimulus was absent in the high-dose chemotherapy group. Basal plasma glucagon concentrations in this group were approximately 3–4 times greater than those of control and were not suppressed following a glucose load. They suggested that cisplatin induces marked glucose intolerance, in association with an impaired insulin response, and an abnormal glucagon response to a glucose stimulus.

Some authors have concluded a fraction of adjuvant chemotherapy regimens produce an appreciable incidence of hyperglycemia [9–13]. To explore this issue, we conducted this study to simulate conditions of a clinical protocol. Chemicals involved in this study included carboplatin (carboplatin group, group 2), paclitaxel (paclitaxel group, group 3), and a combination of carboplatin and paclitaxel (carboplatin-paclitaxel group, group 4). Saline (0.9%) was used as a negative control (group 1). Taking into consideration that some studies [14, 15] have cast doubt on whether dexamethasone contributes to the increased glucose level, as an additional part of the chemotherapy protocol, we set up 4 additional groups, each including dexamethasone plus any of the 4 groups (groups 1′, 2′, 3′ and 4′, respectively).

## Methods

### Animal groups

Eighty clean-grade female Wistar rats, weighing 220 to 280 g, were purchased from the Department of Science of Experimental Animals, Peking University Health Science Center. All animals were housed in static microisolator cage and allowed free access to laboratory chow and distilled water. The 80 rats were weighed and numbered, then were randomized into the following 8 groups: Group 1 (Negative control), Group 1′ (Dexamethasone), Group 2 (Carboplatin), Group 2′ (Carboplatin-plus-dexamethasone), Group 3 (Paclitaxel), Group 3′ (Paclitaxel-plus-dexamethasone), Group 4 (Combined therapy), Group 4′ (Combined-therapy-plus-dexamethasone). Each group comprised 10 rats.

### Chemotherapy

On day 0, the rats in group 1 were treated with 0.9% saline (Beijing Fresenius Cub Medical Co. Ltd., China PR) 2 mg/kg i.v.; group 1′ were treated with dexamethasone (Qilu Pharmacy Co., Ltd., China PR) 1.8 mg/kg i.v.; group 2 were treated with carboplatin (Bristol-Myers Squibb Company, USA) 45 mg/kg i.v.; group 2′ were treated with carboplatin 45 mg/kg and dexamethasone 1.8 mg/kg i.v.; group 3 were treated with paclitaxel (Bristol-Myers Squibb Company, USA) 16 mg/kg i.p.; group 3′ were treated with paclitaxel 16 mg/kg i.p. and dexamethasone 1.8 mg/kg i.v.; group 4 were treated with carboplatin 45 mg/kg i.v. and paclitaxel 16 mg/kg i.p.; group 4′ were treated with carboplatin 45 mg/kg i.v., paclitaxel 16 mg/kg i.p and dexamethasone 1.8 mg/kg i.v.. To imitate chemotherapy in clinic, all doses above were made by formula D-rats = D-human × 0.018 [16]. To mimic the special considerations for the use of paclitaxel, the drug was administered by peritoneal injection twice, each containing half the dose.

### Glucose load

On day 0 (before the chemotherapy), 4, 7 and 14, after fasted for 12 h, the rats in all groups were anesthetized with 2% sodium pentobarbital (Beijing chemical reagent company, Co. Ltd., China PR, 25 ml/kg, i.p.) and treated with 50% glucose, 2 g/kg i.p.. Blood was sampled before and 1 and 2 hours (except day 7 for the poor condition of the rats) after the glucose loading, then reserved for subsequent analysis.

### Blood sample collection

Rats were anesthetized with 2% sodium pentobarbital (25 ml/kg, i.p.), and blood was sampled (0.8–1 ml) from the vena orbitalis posterior. Blood samples were collected in chilled sterilized test tubes containing EDTA (25 μl/ml blood) as an anticoagulant, paclitaxel (5000 U/ml of blood) to inhibit proteolytic degradation of glucagon, and sodium fluoride (4%, 50 μl/ml of blood) as an inhibitor of glycolysis. The samples were preserved at 4 °C for subsequent analysis.

### Blood glucose, glucagon and insulin measurement

Each blood sample was separated into three subgroups for the analysis of glucose, insulin and glucagon through Glucose assay kit (Roche Diagnostics GmbH, Shanghai Company, China PR), Insulin radioimmunoassay kit (Beijing Atom High-Tech Nuclear Technique Utilization Corporation Co. Ltd., China PR) and Glucagon radioimmunoassay kit (Beijing Atom High-Tech Nuclear Technique Utilization Corporation Co. Ltd., China PR) respectively.

### Pathology of pancreas

The Rats in all groups were executed after day 7 or 14, pancreatic tissues were fixed in 4% paraformaldehyde, and paraffin sections were stained with hematoxylin and eosin. Edema, necrosis, inflammation and hemorrhage conditions were measured by Schmidt J score [17].

### Statistics analyze

Homeostatic model assessment (HOMA) indices which shows insulin resistance (HOMA2 IR) and beta cell function percent (HOMA2 %B) were calculated by HOMA-2 calculator [18–20].

Data in the tables and text are expressed as the mean ± standard deviation unless specified otherwise. Between-group comparisons were performed using Kruskal-Wallis one-way analysis. Differences were considered statistically significant at *P* < 0.05. All statistical analyses were conducted using SPSS 20.0 (SPSS Inc., Chicago, IL, USA) and Prism 5 (GraphPad Software, Inc., USA).

## Results

### Glycometabolism in rats treated with chemotherapy

#### Blood glucose level

**Fig. 1:**
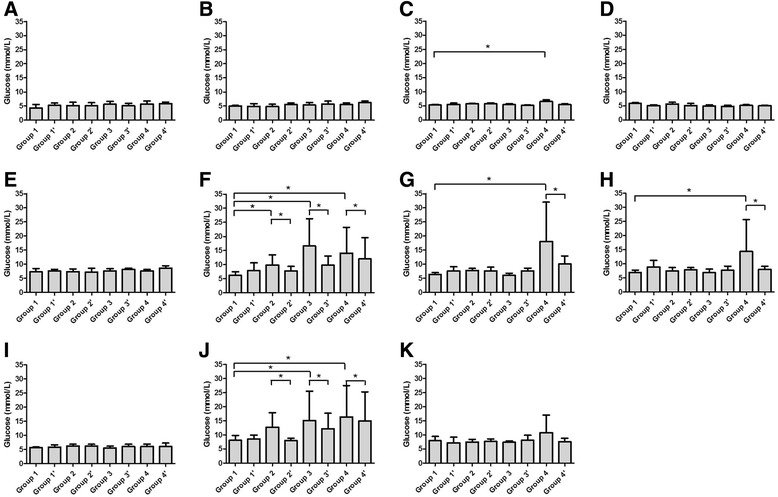
Glucose level of treated rats in eight groups. Glucose levels of rats were measured by hexokinase before feast on day 0(**a**), 4(**b**), 7(**c**), 14(**d**); 1 h after the feast on day 0(**e**), 4(**f**), 7(**g**), 14(**h**); and 2 h after the feast on day 0(**i**), 4(**j**), 14(**k**)

Before the treatment there were no significant difference among 8 groups (*P* = 0.72 for 0 h, *P* = 0.644 for 1 h and *P* = 0.153 for 2hs, Fig. 1a–i). After treatment most of the groups showed a slightly increase of basic glucose level including the negative control. This may be associated with stress induced hyperglycemia during operation (Fig. 1a-d).

There were significant increases in the 0 h glucose level in group 4 on day 7 (*P* = 0.035) after treatment (Fig. 1d).

At 1 h after the loading, increases of glucose levels were observed on day 4 after treatment in group 2, 3 and 4 (*P* = 0.190, 0.008 and 0.025, respectively, Fig. 1f). Increase in group 4 could still be observed on day 7 and day 14 (*P* = 0.045 and 0.278, respectively, Fig. 1g-h).

Changes in glucose level were more aggravated 2 h after loading on day 4 after treatment (Fig. 1j). But all of the changes recovered on day 14, except group 4 (Fig. 1h, k).

The addition of dexamethasone into the chemotherapy protocols was protective, although a slight increase in glucose levels was observed in group 1′ compared with group 1 (Fig. 1f). When used together with chemo drugs, significant decreases were observed in all treatment groups, especially when there was a significant increase in glucose levels after glucose loading (Fig. 1f, j).

#### Plasma glucagon level

**Fig. 2:**
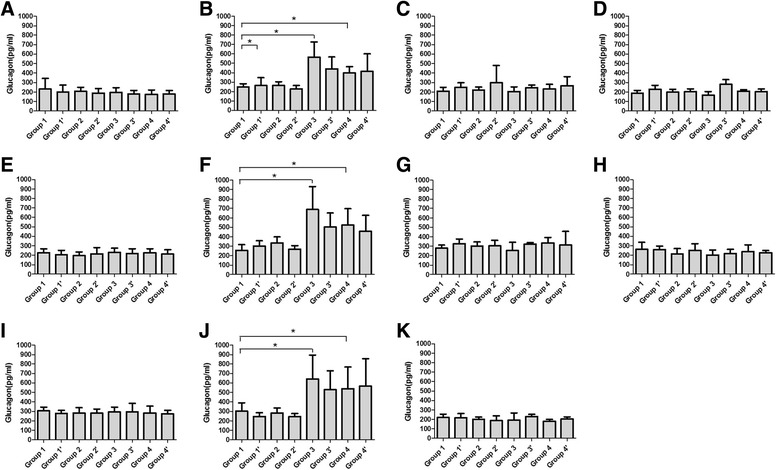
Glucagon level of treated rats in eight groups. Glucagon levels of rats were measured by radioimmunoassay kit before feast on day 0(**a**), 4(**b**), 7(**c**), 14(**d**); 1 h after the feast on day 0(**e**), 4(**f**), 7(**g**), 14(**h**); and 2 h after the feast on day 0(**i**), 4(**j**), 14(**k**)

Basal plasma glucagon concentrations in group 3 were increased on day 4 after treatment (*P* < 0.001, Fig. 2b). This increase was not suppressed following glucose loading (Fig. 2f, j). The increased glucagon level in group 4 was only significantly observed at 1 h on day 4 (*P* < 0.001, Fig. 2f), and these changes just remained for a short period, there was no significance observed on day 7 and 14 after treatment (Fig. G-H, K).

The use of dexamethasone did not increase the levels of glucagon. In contrast, when there was a significant increase in glucagon, such as group 3, the use of dexamethasone seemed to reverse the changes, but not significantly (Fig. 2b–j).

To explore if the increases of glucose level were due to higher level of Glucagon after chemotherapy, we compared these two values simultaneously. In group 1 and 1′, both levels of glucagon and glucose maintain stable with little fluctuation. On day 4, in group 3 and 4, both glucose and glucagon levels increased synchronously, but on day 7 and 14, the synchronization disappeared. This implies that another factor insulin may also plays an important role in the increases of glucose level.

#### Plasma insulin level and HOMA2

**Fig. 3:**
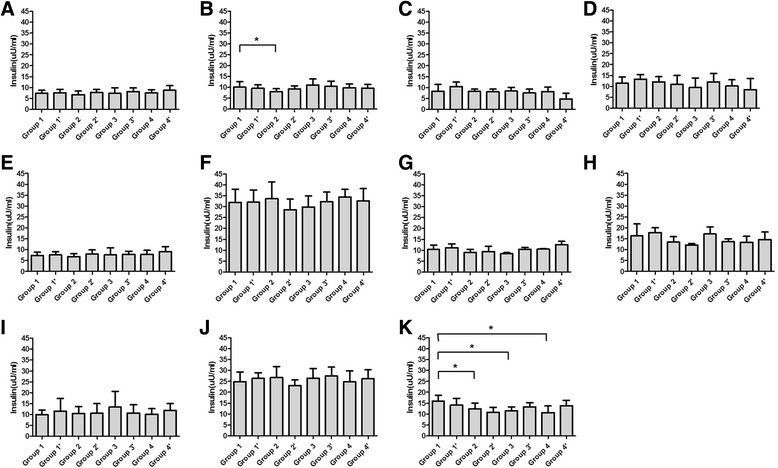
Insulin level of treated rats in eight groups. Insulin levels of rats were measured by radioimmunoassay kit before feast on day 0(**a**), 4(**b**), 7(**c**), 14(**d**); 1 h after the feast on day 0(**e**), 4(**f**), 7(**g**), 14(**h**); and 2 h after the feast on day 0(**i**), 4(**j**), 14(**k**)

The insulin levels without glucose loading before treatment were similar between the groups (*P* = 0.376, Fig. 3a). After treatment, there was a decrease in 0 h insulin levels in group 2 on day 4 (*P* = 0.029, Fig. 3b). There were no differences in 2 h insulin levels between the groups prior to treatment (*P* = 0.726, Fig. 3i). On day 4 after treatment, the 2 h insulin levels of all the 8 groups were increased compared with day 0, but there were no differences between the groups (Fig. 3i, J). On day 14 after treatment, decreases were observed in group 2, 3 and 4 (*P* = 0.043, 0.019 and 0.019, respectively). With regard to 1 h insulin levels, the changes were not as clear as those observed at 2 h. Any differences between the treatment groups were lacking in significance.

The use of dexamethasone did not change the level of insulin significantly. However, when there was a decrease compared with the negative control, the additional use of dexamethasone appeared to eliminate the change.

**Fig. 4:**
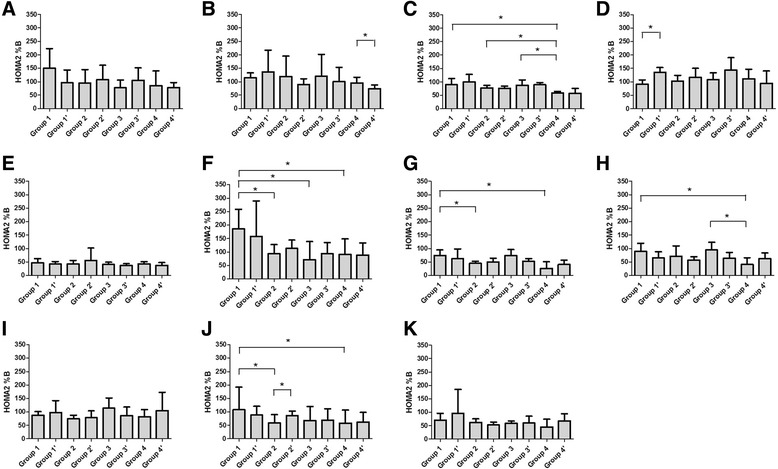
HOMA2 %B of treated rats in eight groups. HOMA2 %B of rats were calculated with blood glucose and insulin by HOMA2 Calculator before feast on day 0(**a**), 4(**b**), 7(**c**), 14(**d**); 1 h after the feast on day 0(**e**), 4(**f**), 7(**g**), 14(**h**); and 2 h after the feast on day 0(**i**), 4(**j**), 14(**k**)

**Fig. 5:**
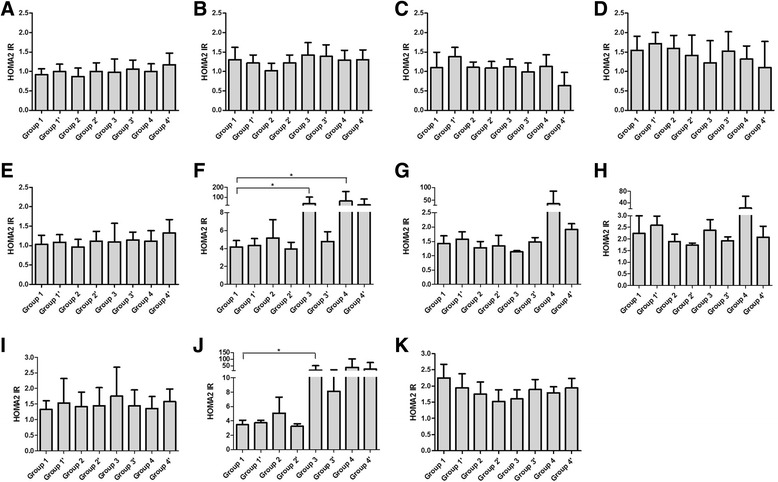
HOMA2 IR of treated rats in eight groups. HOMA2 IR of rats were calculated with blood glucose and insulin by HOMA2 Calculator before feast on day 0(**a**), 4(**b**), 7(**c**), 14(**d**); 1 h after the feast on day 0(**e**), 4(**f**), 7(**g**), 14(**h**); and 2 h after the feast on day 0(**i**), 4(**j**), 14(**k**)

Generally speaking, insulin level goes up and down as a result of glucose changing. But the Insulin levels of groups received chemotherapy seemed not to be correlated with their glucose levels directly (Figs. 1 & 3). Consequently, we observed the change of HOMA2 %B, an index reflects the ability of insulin secretion, in each group. At 1 h after the loading, decreases of HOMA2 %B were observed on day 4 after treatment in group 2, 3 and 4 (*P* = 0.002, 0.002 and 0.006, respectively, Fig. 4f), which was negatively corresponded to the glucose levels (Fig. 1f). Similar trends were also observed at 2 h on day 4, 1 h on day 7 and 14 (Fig. 1g & 4g, Fig. 1h & 4h and Fig. 1j & 4j). HOMA2 IR, another index shows the insulin resistance of rats, significantly increased in group 3 and 4 at 1 h after the loading on day 4 (*P* = 0.034, 0.005 respectively, Fig. 5f). Trends of HOMA2 IR were positively correlated to the glucose levels (Fig. 1g & 5g, Fig. 1h & 5h and Fig. 1j & 4j).

### Survival analysis

**Fig. 6:**
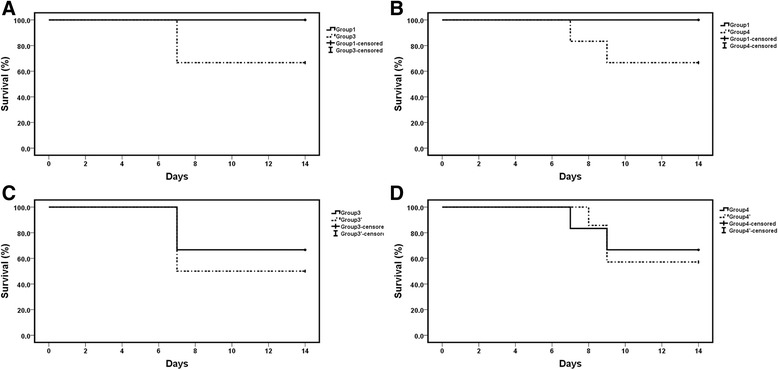
Survival analysis of selected groups. Survival analysis were performed by Kaplan-Meier curve between group 1 and 3 (**a**), group 1 and 4 (**b**), group 3 and 3′ (**c**), group 4 and 4′ (**d**), groups without death were excluded except group 1

In all the 8 groups, the total death number was 10. Six were dead on day 7, one on day 8 and 3 on day 9. There was no dead case in group 1, group 1′, group 2 or group 2′, and death cases were evenly distributed among the other four groups (2 in group 3 and 4, 3 in group 3′ and 4′, respectively). However, as shown in Fig. 6, the overall survival of rats in group 3 was not significantly shorter than that in group 1 (*P* = 0.138), so was it in group 4 (*P* = 0.138). In addition, the use of dexamethasone did not reduce the overall survival in group 3′ (*P* = 0.575, vs group 3) and group 4′ (*P* = 0.817, vs group 4).

### Pathology of pancreases

**Fig. 7:**
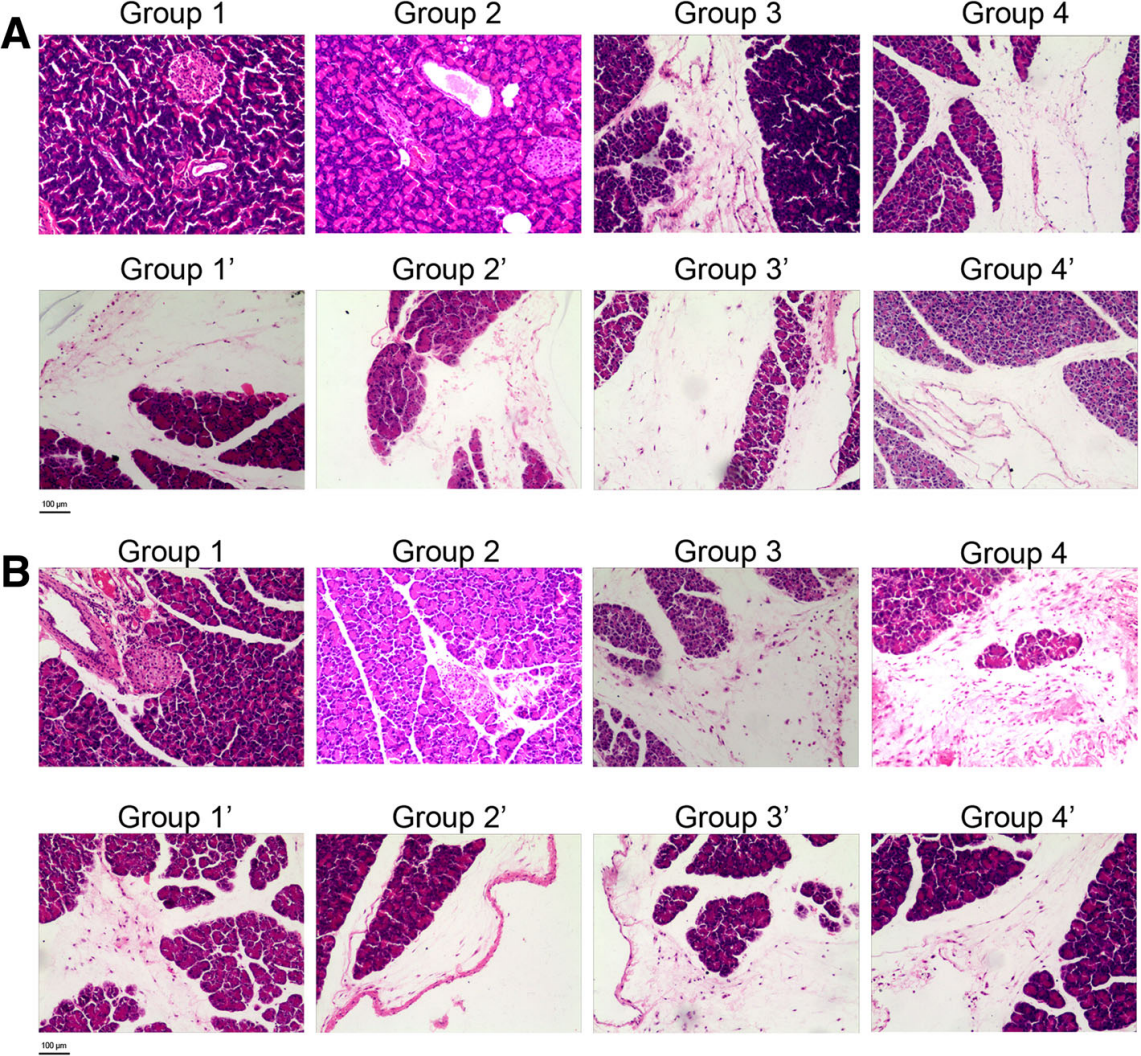
The representative H&E images of the pancreas. **a** Pathology of the pancreas in group 1,1′,2,2′,3, 3′, 4 and 4′ on day 4. **b** Pathology of the pancreas in group 1,1′,2,2′,3, 3′, 4 and 4’on day 14

Necrosis of pancreases cell is almost absent in all of the 8 groups. Hemorrhage and infiltration of inflammatory cells can be observed in every group, but there is no significance among the groups on day 4 (*P* = 0.158 and 0.367 respectively) and day 14 (*P* = 0.073 and 0.052 respectively). The relatively usual change is dropsy, which is localized only in the stroma. The dropsy is more apparently in group 3, 3′, 4 and 4′ on day 4 after treatment (*P* = 0.002, 0.002, 0.000 and 0.000 respectively, vs group 1, Table 1). On day 14 after treatment the edema score decreased, but significant differences could still be observed in group 1′, 2′, 3′ and 4 (*P* = 0.047, 0.043, 0.021 and 0.045 respectively, vs group 1, Table 2). The representative H&E images of the pancreas in all the groups are showed in Fig. 7.

**Table 1:** The pathology scores of pancreases in each group on day 4 after treatment

Groups	Edema (Mean ± SD)	Necrosis (Mean ± SD)	Inflammation (Mean ± SD)	Hemorrhage (Mean ± SD)	Total (Mean ± SD)
Group1	0.00 ± 0.00	0.50 ± 0.58	0.00 ± 0.00	0.25 ± 0.50	0.75 ± 0.50
Group1’	0.50 ± 0.41	0.00 ± 0.00	0.50 ± 0.58	0.00 ± 0.00	1.00 ± 0.91
Group2	0.38 ± 0.25	0.00 ± 0.00	0.50 ± 0.58	0.50 ± 0.58	1.38 ± 1.03
Group2’	0.25 ± 0.29	0.25 ± 0.50	0.25 ± 0.50	0.50 ± 0.58	1.25 ± 1.19
Group3	0.63 ± 0.63 ^*^	0.00 ± 0.00	1.00 ± 0.82 ^*^	0.25 ± 0.50	1.88 ± 1.80
Group3’	0.63 ± 0.48 ^*^	0.25 ± 0.50	1.25 ± 0.96 ^*^	0.25 ± 0.50	2.38 ± 1.25 ^*^
Group4	0.88 ± 0.25 ^*^	0.00 ± 0.00	0.75 ± 0.50	0.25 ± 0.50	1.88 ± 0.85
Group4’	0.88 ± 0.25 ^*^	0.50 ± 0.58	1.75 ± 0.50 ^*^	0.00 ± 0.00	3.13 ± 0.63 ^*^

*P < 0.05, compared with group 1

**Table 2:** The pathology scores of pancreases in each group on day 14 after treatment

Groups	Edema (Mean ± SD)	Necrosis (Mean ± SD)	Inflammation (Mean ± SD)	Hemorrhage (Mean ± SD)	Total (Mean ± SD)
Group1	0.00 ± 0.00	0.00 ± 0.00	0.00 ± 0.00	0.33 ± 0.52	0.33 ± 0.52
Group1’	0.42 ± 0.20 ^*^	0.50 ± 0.55	0.50 ± 0.55 ^*^	0.17 ± 0.41	1.58 ± 0.80 ^*^
Group2	0.08 ± 0.20	0.00 ± 0.00	0.17 ± 0.41	0.67 ± 0.52	0.92 ± 0.49
Group2’	0.50 ± 0.32 ^*^	0.17 ± 0.41	0.33 ± 0.52	0.00 ± 0.00	1.00 ± 0.63 ^*^
Group3	0.13 ± 0.25	0.00 ± 0.00	1.00 ± 0.00 ^*^	0.00 ± 0.00	1.13 ± 0.25 ^*^
Group3’	0.50 ± 0.00 ^*^	0.00 ± 0.00	1.00 ± 0.00 ^*^	0.33 ± 0.58	1.83 ± 0.58 ^*^
Group4	0.38 ± 0.25 ^*^	0.00 ± 0.00	0.25 ± 0.50	0.00 ± 0.00	0.63 ± 0.25
Group4’	0.25 ± 0.29	0.00 ± 0.00	1.00 ± 0.00 ^*^	0.00 ± 0.00	1.25 ± 0.29 ^*^

*P < 0.05, compared with group 1

## Discussion

The side-effects of chemotherapy have been considered as the limitation of quality of life in patients with ovarian cancer. Hyperglycemia is one of the serious side-effects, of which the mechanism is still unclear. It had been demonstrated that cisplatin incorporating paclitaxel as the first-line therapy improves the duration of progress-free survival and of overall survival in women with incompletely resected stage III and stage IV ovarian cancer [21]. Ozols [22] subsequently reported that the combination of carboplatin and paclitaxel could obtain a response equal to that of cisplatin and paclitaxel in the treatment of stage III and stage IV ovarian cancer, with less toxicity. The combination of carboplatin and paclitaxel provided a long-term control of the disease in a great many patients. However, in some cases, hyperglycemia was present during chemotherapy. Some patients even developed diabetes mellitus when treatment had concluded, which may have a negative effect on mortality and morbidity and represented an additional financial burden [23, 24].

The mechanism of hyperglycemia after chemotherapy is to be discovered. The major hypotheses include a defect in insulin secretion, accumulation of glucagon and adjuvant treatment in the chemotherapy protocols. Stress during therapy may also play a role.

Some chemicals have been shown to be associated with beta-cell function damage [24–26]. Wang Y et al. [24] reported that hyperglycemia after cisplatin treatment may be caused by increases in somatostatin and inducible nitric oxide synthase (iNOS) in the pancreatic islets. Wang J et al. [26] demonstrated that increased apoptosis in vivo after chemotherapy and radiation treatment were associated with diabetes mellitus. Our results, in which the HOMA2 %B decreased and the edema score of pancreases increased after the chemotherapy, supported the hypothesis directly, that the hyperglycemia might be caused by the decrease of insulin secretion in pancreatic islets.

On the other hand, the hyperglucagonemia following chemotherapy, which may be related to decreased glucagon degradation associated with impaired renal function [27], contributed to the formation of the hyperglycemia in rats. In our results, the changes of glucagon and glucose before and after chemotherapies were roughly synchronized in group 2 (carboplatin) and 3 (paclitaxel), which implied that the increased level of glucose after treatment could be explained by the decreased glucagon degradation. Furthermore, the changes of HOMA2 IR, an index shows the insulin resistance, were also synchronized with glucose, which indicated that the insulin resistance might be one of the cause of the hyperglycemia too.

Dexamethasone is widely used in chemotherapy; especially in protocols included Paclitaxel, to release side-effects and allergic reaction. Dexamethasone can induce hyperglycemia. But most studies tend to agree that this disorder is minor and temporarily [13, 15]. When treated with dexamethasone (group 1′), only a slim increase of glucose level can be observed compared with group 1 without significance. But in all of the 3 chemotherapy groups, that is group 2 with carboplatin, group 3 with Paclitaxel and group 4 with the combination protocol, the additional use of dexamethasone can reduce the degree of increased glucose levels significantly. The use of dexamethasone does not increase levels of glucagon; oppositely, glucagon levels decreased slightly, especially in group 3. The use of dexamethasone did not change the level of insulin significantly. The reason should be that neither carboplatin nor Paclitaxel induce severe damage on pancreases. These results shows that the protecting on insulin response is a part of the mechanism that dexamethasone could reduce the glucose side-effect of chemotherapy, but not the main one. Further studies are needed to explore this mechanism. The survival analysis shows no difference probably due to the limited use of the chemotherapies. The dose of the drugs was calculated and rats could tolerate it. As a result, the most rats did not die until the terminate day.

## Conclusion

Our result indicates that carboplatin and paclitaxel administration could cause a transient hyperglycemia in rats. This effect may occur by the combination of glucagon accumulation due to the decrease in islet cell secretion. The additional dexamethasone in the combination protocol of carboplatin and paclitaxel does not increase the rats’ blood glucose levels, on the contrary, it seems to reduce the impaired blood glucose metabolism caused by paclitaxel and carboplatin. Multicourse treatment of chemotherapy should be investigated in order to further determine the role of chemotherapy agents in glucose metabolism in rats.

## Abbreviations

D-human: Dose of human
D-rats: Dose of rats
HOMA: Homeostatic model assessment
iNOS: Inducible nitric oxide synthase

## References

[1] Ellis ME, Weiss RB, Korzun AH, Rice MA, Norton L, Perloff M (1986). Hyperglycemic complications associated with adjuvant chemotherapy of breast cancer. A cancer and leukemia group B (CALGB) study. Am J Clin Oncol.

[2] Kelly WK, Curley T, Slovin S, Heller G, McCaffrey J, Bajorin D (2001). Paclitaxel, estramustine phosphate, and carboplatin in patients with advanced prostate cancer. J Clin Oncol.

[3] Belani CP (2001). Interim analysis of a phase II study of induction weekly paclitaxel/carboplatin regimens followed by maintenance weekly paclitaxel for advanced and metastatic non-small cell lung cancer. Semin Oncol.

[4] Fidias P, Supko JG, Martins R, Boral A, Carey R, Grossbard M (2001). A phase II study of weekly paclitaxel in elderly patients with advanced non-small cell lung cancer. Clin Cancer Res.

[5] Weiser MA, Cabanillas ME, Konopleva M, Thomas DA, Pierce SA, Escalante CP (2004). Relation between the duration of remission and hyperglycemia during induction chemotherapy for acute lymphocytic leukemia with a hyperfractionated cyclophosphamide, vincristine, doxorubicin, and dexamethasone/methotrexate-cytarabine regimen. Cancer.

[6] Wang CH, Cui H, Li X, Wang ZQ, Wei LH, Ji XM (2004). Study on factors inducing diabetes mellitus after chemotherapy. *Clin*. J Obstet Gynecol.

[7] Picus J, Schultz M (1999). Docetaxel (Taxotere) as monotherapy in the treatment of hormone-refractory prostate cancer: preliminary results. Semin Oncol.

[8] Goldstein RS, Mayor GH, Rosenbaum RW, Hook JB, Santiago JV, Bond JT (1982). Glucose intolerance following cis-platinum treatment in rats. Toxicology.

[9] Friedland DM, Dakhil S, Hollen C, Gregurich MA, Asmar L (2004). A phase II evaluation of weekly paclitaxel plus carboplatin in advanced urothelial cancer. Cancer Investig.

[10] Feliu J, Martin G, Lizon J, Chacon JI, Dorta J, de Castro J (2001). Sequential therapy in advanced non-small-cell lung cancer with weekly paclitaxel followed by cisplatin-gemcitabine-vinorelbine. A phase II study. Ann Oncol.

[11] Akerley W, Herndon JE, Egorin MJ, Lyss AP, Kindler HL, Savarese DM (2003). Weekly, high-dose paclitaxel in advanced lung carcinoma: a phase II study with pharmacokinetics by the cancer and leukemia group B. Cancer.

[12] Raff JP, Rajdev L, Malik U, Novik Y, Manalo JM, Negassa A (2004). Phase II study of weekly docetaxel alone or in combination with trastuzumab in patients with metastatic breast cancer. Clinical breast cancer.

[13] Graber AL, Porte D, Williams RH (1968). Clinical use of diazoxide and studies of the mechanism of its hyperglycemic effects in man. Ann N Y Acad Sci.

[14] Dispenzieri A, Loprinzi CL (1997). Chemotherapy-induced insulin-dependent diabetes mellitus. J Clin Oncol.

[15] Nan DN, Fernandez-Ayala M, Vega Villegas ME, Garcia-Castano A, Rivera F, Lopez-Brea M (2003). Diabetes mellitus following cisplatin treatment. Acta oncologica (Stockholm, Sweden).

[16] Rosenberg JE, Halabi S, Sanford BL, Himelstein AL, Atkins JN, Hohl RJ (2008). Phase II study of bortezomib in patients with previously treated advanced urothelial tract transitional cell carcinoma: CALGB 90207. Ann Oncol.

[17] Schmidt J, Lewandrowsi K, Warshaw AL, Compton CC, Rattner DW (1992). Morphometric characteristics and homogeneity of a new model of acute pancreatitis in the rat. Int J Pancreatol.

[18] Ehrampoush E, Homayounfar R, Davoodi SH, Zand H, Askari A, Kouhpayeh SA (2016). Ability of dairy fat in inducing metabolic syndrome in rats. SpringerPlus.

[19] Dansuntornwong B, Chanprasertyothin S, Jongjaroenprasert W, Ngarmukos C, Bunnag P, Puavilai G (2007). The relation between parameters from homeostasis model assessment and glycemic control in type 2 diabetes. Journal of the Medical Association of Thailand = Chotmaihet thangphaet.

[20] HOMA-2 calculator www.dtu.ox.ac.uk/homacalculator/. Accessed 15 Mar 2017.

[21] McGuire WP, Hoskins WJ, Brady MF, Kucera PR, Partridge EE, Look KY (1996). Cyclophosphamide and cisplatin compared with paclitaxel and cisplatin in patients with stage III and stage IV ovarian cancer. N Engl J Med.

[22] Ozols RF (1997). Update of the NCCN ovarian cancer practice guidelines. Oncology (Williston Park, NY).

[23] Falkson G, Gelman RS, Pandya KJ, Osborne CK, Tormey D, Cummings FJ (1998). Eastern cooperative oncology group randomized trials of observation versus maintenance therapy for patients with metastatic breast cancer in complete remission following induction treatment. J Clin Oncol.

[24] Wang Y, Aggarwal SK (1997). Effects of cisplatin and taxol on inducible nitric oxide synthase, gastrin and somatostatin in gastrointestinal toxicity. Anti-Cancer Drugs.

[25] Baillargeon J, Langevin AM, Mullins J, Ferry RJ, DeAngulo G, Thomas PJ (2005). Transient hyperglycemia in Hispanic children with acute lymphoblastic leukemia. Pediatr Blood Cancer.

[26] Wang J, Silva JP, Gustafsson CM, Rustin P, Larsson NG (2001). Increased in vivo apoptosis in cells lacking mitochondrial DNA gene expression. Proc Natl Acad Sci U S A.

[27] Goldstein RS, Mayor GH, Gingerich RL, Hook JB, Robinson B, Bond JT (1983). Hyperglucagonemia following cisplatin treatment. Toxicol Appl Pharmacol.

